# Surgical site infection prevention and management in immunocompromised patients: a systematic review of the literature

**DOI:** 10.1186/s13017-021-00375-y

**Published:** 2021-06-10

**Authors:** Federico Coccolini, Mario Improta, Enrico Cicuttin, Fausto Catena, Massimo Sartelli, Raffaele Bova, Nicola de’ Angelis, Stefano Gitto, Dario Tartaglia, Camilla Cremonini, Carlos Ordonez, Gian Luca Baiocchi, Massimo Chiarugi

**Affiliations:** 1 General, Emergency and Trauma Surgery Department, Pisa University Hospital, Via Paradisa 1, 56100 Pisa, Italy; 2 General Surgery Department, Bologna University Hospital, Bologna, Italy; 3 Emergency Surgery Department, Parma University Hospital, Parma, Italy; 4 General Surgery Department, Macerata Hospital, Macerata, Italy; 5 Unit of Digestive and Hepato-biliary-pancreatic Surgery, Henri Mondor Hospital, Créteil, France; 6 UPEC, University Paris Est, Créteil, France; 7 Department of Experimental and Clinical Medicine, Firenze University, Firenze, Italy; 8 Department of Surgery, Fundación Valle del Lili, Cali, Colombia; 9 Department of Clinical and Experimental Sciences, University of Brescia, Brescia, Italy

**Keywords:** Infection, Immunity, Wound, Care, Costs, Drugs, Inflammatory

## Abstract

**Background:**

Immunocompromised patients are at higher risk of surgical site infection and wound complications. However, optimal management in the perioperative period is not well established. Present systematic review aims to analyse existing strategies and interventions to prevent and manage surgical site infections and other wound complications in immunocompromised patients.

**Methods:**

A systematic review of the literature was conducted.

**Results:**

Literature review shows that partial skin closure is effective to reduce SSI in this population. There is not sufficient evidence to definitively suggest in favour of prophylactic negative pressure wound therapy. The use of mammalian target of rapamycin (mTOR) and calcineurin inhibitors (CNI) in transplanted patient needing ad emergent or undeferrable abdominal surgical procedure must be carefully and multidisciplinary evaluated. The role of antibiotic prophylaxis in transplanted patients needs to be assessed.

**Conclusion:**

Strict adherence to SSI infection preventing bundles must be implemented worldwide especially in immunocompromised patients. Lastly, it is necessary to elaborate a more widely approved definition of immunocompromised state. Without such shared definition, it will be hard to elaborate the needed methodologically correct studies for this fragile population.

## Background

Surgical site infection (SSI) is a global health issue [1, 2]. Both general and emergency surgery are burdened by a high rate of SSI in immunocompetent patients (IP) [3–6]. Immunocompromised state, in fact, and wound healing-impairing drugs assumption additionally increase the risk of infection. Reported SSI rate among transplanted patients is up to 27% and up to a quarter of them may need an interventional (or surgical) procedure [7–9]. In 2016, the World Health Organization (WHO) released guidelines on SSI prevention [2]. While some interventions aiming to reduce SSI are shared and accepted (i.e. antiseptic surgical prep, perioperative antibiotic prophylaxis) others lack definitive evidence. The use of negative pressure wound therapy (NPWT vs. standard dressing (SD)) for example, was suggested with a low quality of evidence for high-risk patients. It has been proposed that when NPWT is applied to the surgical incisional wound with closed suture (iNPWT), it would reduce the rate of SSI, especially in a high-risk population, and some series also individuated a benefit in the acute care surgery [10, 11]. iNPWT is usually not considered harmful, but it is expensive compared to SD, therefore accurate evaluation of the cost-benefit balance is needed.

Other wound management and dressing techniques have been evaluated in the literature trying to reduce SSI incidence, but no definitive results have been obtained. Moreover, management of perioperative steroids, immunomodulatory drugs and additional “wound-healing” impacting medication is far from being fully understood [2, 12, 13]. Lastly, transplanted patient carries peculiar challenges given the acquired immunocompromised condition which can be only partially modulated from the treating physician. This review aims to address the best intervention to optimize wound management and minimize complications in general and emergency surgery in immunocompromised and high-risk patients.

Basing on the existing literature, five principal areas of interest were investigated: (1) specific intervention in transplanted patients and in patients under immunomodulatory therapy undergoing surgery, (2) perioperative management of drugs affecting wound healing/SSI, (3) oncological patients operated under chemotherapy, (4) wound dressing in immunocompromised patients, and (5) high-risk patients undergoing general or emergency surgery.

## Materials and methods

This systematic review was conducted based on PRISMA methodology [14]. SSI was identified as the primary outcome. SSI was defined according to the World Health Organization classification: Surgical site infection is also defined as an infection that occurs within 30 days after the operation and involves the skin and subcutaneous tissue of the incision (superficial incisional) and/or the deep soft tissue (for example, fascia, muscle) of the incision (deep incisional) and/or any part of the anatomy (for example, organs and spaces) other than the incision that was opened or manipulated during an operation (organ/space) [15].

### Definition of the immunocompromised patient

An immunocompromised host is a patient presenting an impaired or weakened immune system; this does not allow a normal response to infections.

Immunocompromised patients are defined as follows [16, 17]:

1. Congenital conditions (T- or B-cell defects, macrophage dysfunctions, often in newborns and children but even in the adult population)
2. Acquired conditions:
  - Infected by human immunodeficiency virus (HIV) who developed acquired immunodeficiency syndrome (AIDS)
  - Hematologic malignancy
  - Patients affected by intrinsic immune conditions considered immunodeficiency along with one between “solid malignancy or solid organ transplanted patients or inflammatory disease/rheumatologic disease” plus the concurrent assumption of immunomodulatory drugs or chemotherapy
  - Patients in a physiologic or pathologic condition that is accompanied by any degree of immunodeficiency

#### High-risk population

Beside the properly defined immunocompromised patients, many other ones present a mix of conditions, surgical risk factors, and physiological states which increase the risk of SSI and contribute to define the high-risk population [16]. These conditions may be listed as follows:

- *Patients conditions*. Low serum albumin concentration, older age, obesity, smoking, diabetes mellitus, and ischemia secondary to vascular disease or irradiation
- *Surgical risk factors.* Prolonged procedures and inadequacies in either the surgical scrub or the antiseptic preparation of the skin
- *Physiological states*. Trauma, shock, blood transfusion, hypothermia, hypoxia, and hyperglycemia

Included trials are those about IP and high-risk populations analysing benefit of iNPWT over SD or other dressing/management techniques, techniques for suturing the surgical incision, and the effect of steroids, immunomodulatory, or other drugs affecting wound healing and infection development.

### Search strategy and articles inclusion

A systematic search was conducted from January 2000 to March 2020, for all articles on immunocompromised patients and surgical site infection in MEDLINE via PubMed, The Cochrane Library, and Scopus by two reviewers (FC, MI) independently. The following terms were used: surgical site infection; SSI; immunocompromised; immunosuppressed; wound; primary, secondary closure; iNPWT, combined with AND/OR. Given that definition of immunocompromission is wide and there is no consensus, the search included also terms as “HIV”, “AIDS”, “transplanted”, and “chronic steroid therapy” with synonyms and MeSH terms. Manual and reference-text research for additional relevant studies was allowed. All duplicates, articles in a language other than English, and animal studies were removed. Abstracts were screened and not relevant studies were removed; then, full-text assessment of the articles was performed. Randomized controlled trials (RCTs), meta-analysis, prospective, and retrospective studies that included rate of SSI as the first outcome, length of hospital stay (LOS), and other surgical complications were included. Studies with surgical specialties other than general and colorectal (orthopaedic, vascular, spinal, neurosurgical, obstetrics, thoracic, and cardiac) were excluded, unless they specifically deal with immunocompromised patients.

Studies on SSI in adult population (> 18 years old) in general surgery where high-risk patients were assessed even if there was no formal definition of immunocompromised state were included (i.e. oncologic patients, diabetic with other comorbidities, patients on immunosuppressant). Case reports were excluded. Case series with less than 30 patients were also excluded unless they reported relevant results (e.g. given the high number of RTCs on iNPWT, small retrospective (< 30 patients) studies on the same topic were excluded, conversely small “unique” RCTs on anti-vascular endothelial growth factor (VEGF) therapy were included).

In case of disagreement between the two senior reviewers (FC, MI), the consensus was reached by discussion, if there was no consensus a third reviewer was sought (FCa).

**Fig. 1:**
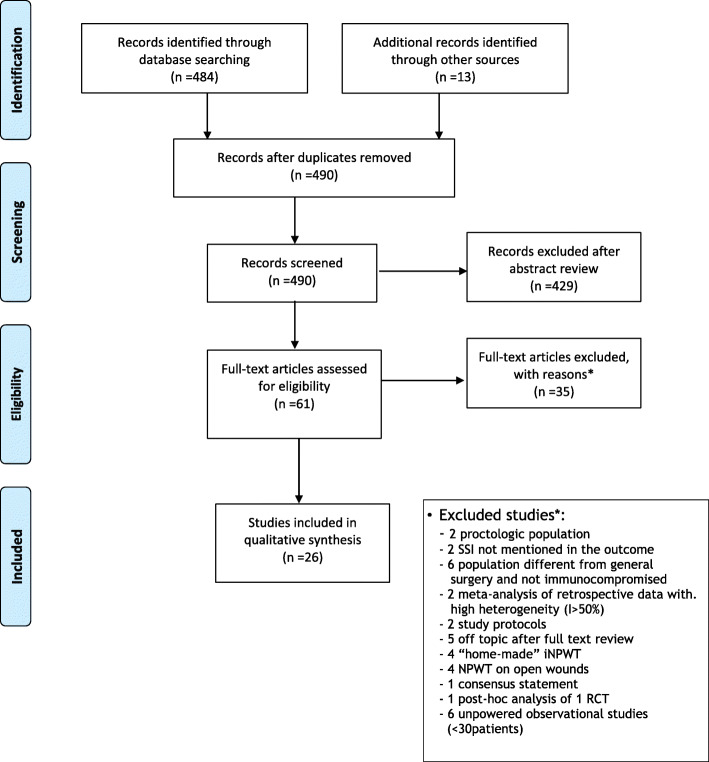
PRISMA flow chart for research results

Research results were reported according to PRISMA flow chart in Fig. 1.

## Results

There is a paucity of studies focusing on immunocompromised patients and specifically on SSI prevention and management. Relevant studies to be included are listed in Table 1. Twelve were RCTs, 6 meta-analysis, 4 retrospective studies, 1 post-analysis of pooled data from RCTs, one systematic review, one review of RCTs, and one prospective study. One post-analysis of RCTs was included due to its unicity and its ability to predict the outcome in the specific population (SSI in patients on immunomodulatory drugs) [12]; similarly, one systematic review with analysis of case series/report was admitted since it was focused on the topic of the study [18]. Some trials presented heterogeneous cohorts of patients, with a percentage of them formally definitely as immunocompromised. In some studies, immunocompromised state was deducible from other data (patients on chronic steroid therapy, or patients on immunomodulatory drugs or patients with diffuse metastatic cancer undergoing chemotherapy), these studies were as well included in the analysis. The quality of evidence is not homogeneous for all the topics and consequently definitive recommendations about all issues cannot be given. Summary of the key-points in SSI prevention and management is given in Table 2.

**Table 1:** Summary of the included studies (*RCT* randomized controlled trial, *SSI* surgical site infection, *iNPWT* negative pressure wound therapy, *SD* standard dressing, *Cht* chemotherapy, *BMI* body mass index, *IFX* infliximab)

Topic	Author	Year	Study type	Intervention	Setting	Number of patients	N. of IP	Results
**Transplanted patients**	Dean P.G.	2004	RCT	Sirolimus vs tacrolimus	Kidney transplant	123	All	Higher rate of SSI in sirolimus
	Siskind E.	2012	Prospective	Partial incision closure	Kidney transplant	104	All	No SSI
	Shahrestani S.	2018	Meta-analysis	Sirolimus, BMI, different surgical incisions	Kidney and pancreas transplant	17821	All	Higher rate of hernia in sirolimus
	Gurusamy	2014	Review of RCTs	Bowel decontamination, Pre- and probiotics, G-CSF	Liver transplant	614	All	No difference in SSI or complication rate
	Shrestha M.S.	2016	Systematic review	NPWT for complication	Kidney transplant	22	All	Heterogeneous
	D’Souza K.	2019	Syst rev. of retrospective	Drain vs. no drain	Kidney transplant	1640	All	No difference in SSI or complication rate
	Berry	2019	RCT	72 h-long antibiotic prophylaxis vs intraoperatory antibiotic alone	Liver transplant	97	All	
**Colorectal cancer**	Kabbinavar F.	2005	RCT	CHT +/− bevacizumab	Metastatic colorectal cancer	209	All	/
	Hurvitz H.	2004	RCT	CHT +/− bevacizumab	Metastatic colorectal cancer	813	All	/
	Scappaticci F.A.	2005	Pooled data from RCT	CHT +/− bevacizumab	Metastatic colorectal cancer	1132	All	SSI: 13% BZ+CH vs 3.4% CH alone
	Curran T.	2018	Retrospective	iNPWT vs SD	High-risk open colorectal surgery	315	61 (chronic steroid/metastatic cancer)	SSI: 7% iNPWT vs 15% SD
**Crohn disease**	Bafford A.C.	2013	Retrospective	Patients on immunomodulatory therapy	Crohn disease	196	127 (on drugs)	Same rate of SSI
	Canedo J.	2010	Retrospective	Patients on IFX, other drugs or assuming no drugs	Crohn disease	225	150 (IFX or other drugs)	No difference in SSI
**Trauma**	Costa M.L.	2020	RCT	iNPWT vs SD	High-risk patients	1629	Not specified	No difference in SSI rate
	Masden D.	2012	RCT	iNPWT vs SD	High-risk patients	81	7	No difference in SSI rate
**Mixed High-risk population**	BlackHam A.U.	2013	Retrospective	iNPWT vs SD	Abdominal oncological surgery	191	76 (neoadjuvant cht)	SSI: 6.7% iNPWT vs 19.5% SD
	Javed A.A.	2019	RCT	iNPWT vs SD	High-risk pancreatico-duodenectomy	123	77 (neoadjuvant cht)	SSI: iNPWT *9%* vs 31.1% SD
	Murphy P.B.	2019	RCT	iNPWT vs SD	Open colorectal	288	9	No difference in SSI rate
	O’Leary D.P.	2017	RCT	iNPWT vs SD	Abdominal surgery	49	Not specified	SSI: iNPWT *8.3%* vs 32% SD
	Li P.-Y.	2017	RCT	iNPWT vs SD	Abdominal, colorectal surgery	71	Not specified	SSI: iNPWT *3%* vs 23.7% SD
	Shen P.	2017	RCT	iNPWT vs SD	Abdominal, oncological surgery	265	Excluded	No difference in SSI rate
**Mixed High-risk population**	Strugala and Martin	2017	Meta-analysis (RCT + observational)	iNPWT vs SD	All specialities	1863	Not specified	SSI: iNPWT 4.8% vs 9.7% SD
	Zwanenburg P.R.	2019	Meta-analysis (RCT + observational but only RCT reported)	iNPWT vs SD	All specialities + subgroup analysis	4398	Not specified	No advantage in NPWT if stratified for surgical specialties
	Kuper T.M.	2020	Meta-analysis of RCTs	iNPWT vs SD	Open abdominal	792	Not specified	No difference in SSI rate
	Sahebally S.	2018	Meta-analysis (RCT + observational)	iNPWT vs SD	Open abdominal	1187	Not specified	NPWT > SD pooled OR 0.25

**Table 2:** SSI prevention and management key-points (*SSI* surgical site infection, *VEGF* vascular endothelial growth factor, *iNPWT* incisional negative pressure wound therapy)

Topic	Key-points
**General consideration**	**Suggestion:** the very first and fundamental step to reduce SSI rate is the improving of adherence to SSI care bundles and guidelines at all steps of patient management.
**Immunosuppressant and wound healing-impairing therapy**	**Suggestion:** immunosuppressant therapy must be carefully evaluated in a multidisciplinary approach in the event of emergency or elective surgery. **Suggestion:** immunosuppressant therapy adjustment should be taken into account in the perioperative period in emergency or elective surgery in transplanted patient. **Suggestion:** mTOR sparing regimen may be considered in the perioperative period. **Suggestion:** in patients under mTOR inhibitors treatment, it may be considered to switch to a calcineurin inhibitor or other immunomodulatory regimens in the perioperative period. **Suggestion:** in emergent procedures on patients currently assuming drugs that may affect wound healing or SSI rate (anti VEGF, steroids, etc.), an accurate and balanced multidisciplinary plan for therapy and surgery is mandatory.
**Negative pressure wound therapy**	**Suggestion:** iNPWT have shown no harm but it is scarcely effective in reducing SSI in immunocompromised and high-risk patients undergoing surgical procedures. **Suggestion:** iNPWT may be considered an option to treat or prevent wound complications after solid organ transplant.
**Skin closure**	**Suggestion:** partial skin closure with interrupted stitches is a feasible option to reduce SSI in transplanted patients. This option should be considered even in the event of emergency abdominal surgery in immunocompromised patients. **Suggestion:** early definitive skin closure should be considered once the risk of SSI has been reasonably cleared out.
**Surgical drain**	**Suggestion:** surgical drains placement in transplant patients seems to not influence the wound complication rate.

As said however, under the category of immunocompromised patient, or even under the definition of patient at high risk of developing SSI, we found a myriad of different specific conditions that have been differently investigated by literature.

Given this characteristic of the research, the systematic review of literature enlightens specific answers to specific questions, leaving some areas uncovered. At present, from literature, it is possible to obtain precise data about the following topics, while the general frame can be only deducted by interpretating specific suggestions.

In transplanted patient, precise data exist regarding the following:

- Role of prophylactic antibacterial therapy and preventive methods
- Immunosuppressant therapy and SSI in kidney transplant
- Skin closure and SSI in kidney transplant
- Surgical drain and SSI in kidney transplant

Regarding drugs that may affect wound healing literature explores:

- Oncological patients under chemotherapy and SSI
- Inflammatory bowel disease under immunomodulatory therapy and SSI

The effect of incisional negative pressure wound therapy has been investigated in the following conditions:

- iNPWT and SSI in transplanted patients
- iNPWT and SSI in patients under immunomodulatory drugs
- iNPWT and SSI in high-risk patients

### Transplanted patients

#### General considerations

SSI in transplanted patients carries several consequences, with a consistent increase in graft loss rates, morbimortality, LOS, and costs [19–21]. Considering liver, kidney, and pancreas transplant, some risk factors have been indicated. Considering the peculiar aspects related to the transplantation, the most present risk factors, according to different studies, are intraoperative red blood cells transfusion, prolonged cold ischemic time, necessity of reintervention, and extended criteria donor-related transplant [20–28]. Individual surgical technique and practice could also be related to SSI risk [19, 29].

#### Methods to prevent SSI in transplanted patients

Gurusamy et al. [30] presented a review of RCTs, with the aim to assess the efficacy of different methods use to prevent infective complication, systemic, and site-related, in liver transplanted patients. Examining seven RCTs with 614 patients, the review was unable to recognize significant benefits in using selective bowel decontamination, use of prebiotics with probiotics, use of prebiotics, and granulocyte-colony stimulating factor (G-CSF), neither according to mortality rate nor to re-transplantation. Selective bowel decontamination, compared to prebiotics with probiotics, could even increase the rate of infection.

#### Antibacterial prophylaxis in transplantation

The role of antibiotic prophylaxis in transplantation is strongly debated, especially considering the increasing emergence of multidrug-resistant bacteria, and its utility is questioned [28, 31–33]. Berry et al. [34], in a randomized controlled trial, compared intraoperative antibiotic prophylaxis alone against perioperative 72-h-long prophylaxis in liver transplantation. The study showed no differences in the primary outcome of 30-day SSI rates in both intention-to-treat (19% versus 27%; *P* = 0.39) and per-protocol (23% versus 24%; *P* = 0.87) analyses between the two groups. Furthermore, no significant differences in multiple secondary outcomes were recorded.

#### Immunosuppressant therapy and SSI

A randomized trial on kidney transplanted (KT) patients investigated the rate of SSI in patients treated with a sirolimus- or tacrolimus-based therapy [35]. Both groups received prednisone daily for 1 month. Wound complication rate (infection, incisional hernia, and dehiscence) was higher in the sirolimus group (47% vs 8%, p < 000.1) [35]. This difference was even more evident in obese patients. A recent meta-analysis demonstrated a higher rate of incisional hernia (3.7 to 18.1% (p < 0.001)) and wound complications in patients treated with mammalian target of rapamycin (mTOR) inhibitors (e.g. Sirolimus) compared to mycophenolate mofetil [13].

#### Skin closure and SSI

A recent prospective observational study investigated 104 patients with their incision closed “partially” with interrupted stitches at 4 cm apart from each other with no drains placed. Patients were permitted to shower at postoperative day one. No patient experienced SSI infection either immediately postoperatively or at follow-up. Patient satisfaction scores ranked up to 99% [36].

#### Surgical drain and SSI

It is unclear if the placement of surgical drainage in KT lessens or increases the SSI rate. The rationale would be the reduction of deep fluid collections and the access to prompt diagnosis in the event of complications [37]. No randomized trials exist addressing the topic. A recent meta-analysis of retrospective studies investigated a total of 1640 patients undergoing KT of whom 1023 had drain and 617 had not [38]. Patients on mTOR inhibitors were excluded in all studies but one. Drain insertion reduced the risk of deep fluid collections without a decrease in risk for wound complications.

### Drugs that may affect wound healing

Some drugs may impair wound healing, an effect that could be enhanced in the immunocompromised population. Oncological patients undergoing surgery while in therapy with VEGF inhibitors (i.e. bevacizumab) may be exposed to higher surgical site events. Patients affected by inflammatory bowel disease (IBD) may assume drugs that impair wound healing and may favour surgical site complications.

#### Oncological patients under chemotherapy and SSI

Data from two randomized trials [39, 40] were analysed to assess the influence of bevacizumab on wound healing in urgent or emergent surgical procedures. Authors extracted data from the trials creating two cohorts: the first, patients who underwent surgery during VEGF-inhib. suspension and 28 to 60 days before restarting treatment with VEGF-inhib., and in the second, patients who underwent surgery during treatment. The authors found a complication rate (including dehiscence, fistula formation) of 3.4% when bevacizumab was administered more than 28 days after surgical intervention, and a complication rate of 13% in patients who underwent surgery during treatment; no statistically significant difference was found at the analysis.

The long half-life of the bevacizumab (up to 20 days) must be considered. Its immediate suspension in face of emergency surgery could not reduce its circulating level on time [41]. The suspension should be accurately evaluated with the oncologist in a multidisciplinary and tailored risk-benefit balance.

#### Inflammatory bowel disease under immunomodulatory therapy and SSI

A retrospective analysis of patients undergoing surgical procedures for Crohn’s disease (CD) [42] compared patients not taking any immunomodulatory therapies with patients on immunomodulatory drugs. Patients taking medications received 127 procedures, with a 37% of them taking steroids. No difference in the rate of SSI were found between the two groups suggesting that steroids may not affect SSI rate in the postoperative period (overall incidence was 9.2%). However, these results may be biassed since “no-medication” group had a higher rate of urgent surgical procedure (27.6% vs. 13%, p = 0.02) than patients on steroids. The urgent setting may have increased the amount of complication in the “non-steroid” group. Emergency surgery was associated with a complication rate of 28.9 vs 20.5% in the elective surgery group (not statistically significant).

A retrospective cohort trial showed as perioperative administration of infliximab or steroids may not be associated with increased morbidity if compared to their non-administration in patients with CD undergoing surgical procedure [43]. However, the two groups were not homogenous. In fact, patients in the group where these drugs were not administered were older, with higher rate of comorbidity, lesser rate of laparoscopic approach, and more previous surgical intervention.

### Incisional negative pressure wound therapy

#### iNPWT and SSI in transplanted patients

A recent systematic review analysed the feasibility of iNPWT for the treatment of wound complications in KT [18]. A total of 22 cases were retrieved from the international literature and analysed. NPWT was successfully used to treat wound complications (ranging from wound dehiscence to urine leak). Conflicting results exist regarding the use of NPWT in post-KT urinary leak [44, 45]. Pre-emptive NPWT was applied in a series of 9 patients undergoing KT: no SSI or other postoperative wound complication were observed [46].

No literature exists about the application of NPWT in patients undergone to other solid organ transplant. However, it is reasonable to consider its possible efficacy in reducing postoperative wound complication in all the patients undergone to solid organ transplant other than kidney.

#### iNPWT and SSI in patients under immunomodulatory drugs

A retrospective analysis of high-risk patients (including patients on immunomodulatory drugs or steroids) investigated the benefit of iNPWT [47–49]. Patients were matched in two groups using the surgical site infection risk score (SSIRS), that aims to predict the SSI probability according to patient’s comorbidities and physical factors [50]. The predicted risk for the standard dressing group was almost 19%, while the predicted risk for the iNPWT group was 20%. The study found and overall SSI rate of 13% reduced to 6.5% in the iNPWT group (P = 0.05). Patients with iNPWT more frequently resulted to have a stoma (92% vs. 48%).

#### iNPWT and SSI in high-risk patients

An RCT (The P.I.C.O. Trial) analysed 50 patients undergoing general or emergency abdominal surgery whose incisions were treated with or without iNPWT [51]. A beneficial effect of iNPWT over SD with the max effect manifesting at 30 days post operatively (8.3% vs 32%, p = 0.043). No difference in SSI rate was observed at postoperative day 4 (the day chosen for the first undressing). The trial was limited to a small sample size of 50 patients and presents the lack of blinding in the SSI assessment. Another RCT focusing on colorectal procedures randomized 71 patients either to iNPWT or SD (93% of the population presented a clean-contaminated surgical fields) [52]. Overall incidence of SSI was 14% and iNPWT significantly decreased the incidence of SSI (3% vs 23.7%, p = 0.03). In 2017, a RCT was published, focusing on pancreatic, gastrointestinal, and peritoneal surface oncologic procedures where immunocompromised patient and those on steroid were excluded [53]. There was no difference in incidence in the rate of SSI or wound dehiscence for the patients in the iNPWT group or the SD group. (12.8% vs 12.9%, p > 0.99); even when patients were stratified by type of surgery, the absence of difference persisted.

The same study group presented a retrospective analysis with different results where they observed reduction of SSI with iNPWT vs. SD (6% vs. 27.4%, p = 0.001) [54].

An RCT analysed 123 high-risk patients allocated to receive either iNPWT or SD after pancreatic surgery [55]. The overall SSI rate was 15–20%, which is lower compared to the existing literature in this population. To select the population in which iNPWT would best benefit, they individuated an “high risk SSI” population according to SSIRS (patient who had received neoadjuvant chemotherapy, biliary stenting or both) [56]. They found 9.7% of SSI in the iNPWT group versus 31.1% in the control (P = 0.003).

A randomized trial on 284 patients (the NEPTUNE Trial) compared SD vs. iNPWT in open colorectal procedures. No difference was found in the 30 days SSI rate in the two groups (32% vs 34%, P = 0.68). 9.3% of patients were either immunocompromised or on chronic steroid therapy, and they were equally distributed through the two groups [3].

Two large multicenter randomized trials on trauma population undergoing surgery for lower limbs fractures found no difference in SSI rate in patients treated with iNPWT vs. SD [57, 58]. In one of the two trials, 8% of patients were immunocompromised. There were no patients with abdominal wounds treated with iNPWT since the majority of wounds were in the legs.

The great discordance between results of these trials inspired several meta-analyses. However, in some of these papers, immunocompromised and high-risk patients were mixed together with other cohorts. Indicative results may be obtained from those meta-analyses; however, specific indications cannot be finalized.

Strugala et al. took into account several papers on spinal, orthopaedic, breast, and vascular surgery [59]. They observed a reduction in SSI from 9.7 to 4.8% when iNPWT was used. The baseline rate of SSI (9.7%) is very different from the baseline rate of abdominal emergency surgical intervention. When the analysis was conducted on observational studies instead of on RCTs, the SSI rate in SD group was 22.5% versus 7.4% in iNPWT one: the difference in SSI rate in the SD groups between retrospective and RCTs was 22.5% vs. 9.7% respectively. Besides, when this meta-analysis was published, several RCTs now available were lacking.

Another meta-analysis focused on iNPWT in colorectal and abdominal elective and emergency surgery [60]. Researchers found significant reduction of SSI when using iNPWT also if only colorectal procedures were analysed. No effect of iNPWT on seroma and wound dehiscence rate was found. When this meta-analysis was made, the NEPTUNE trial [3] was ongoing; therefore, their results did not enter the pooled analysis; additionally, their results were mainly based on retrospective studies. Three RCTs were included in the meta-analysis and no RCTs entered the specific analysis for the colorectal procedures alone; no specific focus on immunocompromised patients was done.

In 2019, Zwanenburg et al. conducted a meta-analysis with a meta-regression taking into account the wide heterogeneity within the RCTs. They found that iNPWT decreased SSI rate. They did not stratify results for various surgical categories, but they did it for surgical field conditions (i.e. clean, clean contaminated) [61]. In the same paper, subgroup analysis focusing on abdominal surgery was performed: the benefit of iNPWT over SD was no longer highlighted.

Lastly, Kuper et al. [62] conducted another meta-analysis and found no difference in the outcome between iNPWT and SD — with high heterogeneity between patient groups.

## Discussion

The definitions of the IP are multiple and heterogeneous [63]. Several disease and drug regimens result as immunocompromising; moreover, immunocompromised condition is dynamic with a large variability in severity even within the same patient’s life [64].

Reducing SSI in elective and emergency abdominal surgery has always been matter of great attention. This is exceptionally true in the IP where SSI morbidity and mortality are higher.

A prompt recognition of the IPs allows to insert them in a high-risk category and, thus, to establish the correct preventive measures. However, as we can extract from the transplantation experience [30], it is difficult to recognize precise methods to start reducing the risk of SSI before the intervention. Even considering that only one trial was at low risk of bias, this review of RCTs showed that practices such as bowel decontamination, administration of prebiotics and probiotics, use of granulocyte-colony stimulating factor (G-CSF) could be of scarce advantage, in terms of mortality, re-transplantation, and hospital stay. The use of bowel decontamination, compared to the administration of pre- and probiotics, is characterized by an increase rate of infections.

In the same time, commonly used practices like antimicrobial prophylaxis could be inefficient to prevent SSI in IPs [31–33]. Furthermore, misuse of antibiotics is burdened by increasing rates of selection of multidrug-resistant bacteria that could be lethal for any IP. As Berry et al. [34] demonstrated with their investigation, any daily practice should be questioned in a critic optic, in order to maximize the desirable benefits, reducing risks and abuses. Their study analysed the differences in SSI rates between liver transplant recipients who received extended antibiotic prophylaxis (until 72 h from the procedure) compared to the patients who received a single, intraoperatory dose of antibiotic. Considering a declared power of 60%, the study showed no differences in terms of SSI, nosocomial infection, time to infection, intensive care unit stay, and cumulative hospital stay. These results clearly indicate the necessity to question the real efficacy and rationale of our prophylactic choices and also the need for further randomized studies about this complex topic. Ultimately, this could lead also to a proper understanding of the role of prophylactic measures in IPs: at present, no indication about a shortening of antibiotic prophylactic therapy can be given, and the authors suggest to act according to the general guidelines on the topic, with an eye on the local most common pathogens involved in SSI and a special attention to the fragility of the patient.

WHO 2016 guidelines on SSI [2] gave a conditional recommendation with low quality of evidence regarding iNPWT: “suggested on primarily closed surgical incisions in high-risk wounds, to prevent SSI, while taking resources into account”. At the time of recommendation production some RCTs were not available (e.g. The NEPTUNE trial [3]); moreover, WHO statement considered the entire surgical populations, not focusing on IP.

One year later, the WHO guidelines, in an international multidisciplinary consensus group [65], elaborated some recommendations about the use of iNPWT. The authors suggested that high-risk patients undergoing high-risk procedures should have iNPWT. Unfortunately, the vast majority of RCTs included did not focalize on the abdomen as primary site for SSI.

In the present systematic review, 7 RCTs, 2 meta-analysis, and 2 retrospective studies on iNPWT were included. Four RCTs found no benefit with iNPWT while 3 found an advantage; 2 meta-analysis found benefit in iNPWT, while two found no benefit at all. In retrospective studies, data on the incidence of SSI in standard dressing groups or iNPWT did not match the RCTs results. The reason for these differences, especially when found in RCTs must be investigated.

Some RCTs or meta-analysis evaluated a mixed and different population from the one matter of the present study as trauma patients [38] or other surgical specialties (vascular, breast, gynaecological neoplastic patients) [52, 58]. In subgroup analysis performed for abdominal surgery iNPWT benefit disappeared. In included trials, different approaches to postoperative dressing in the iNPWT and the SD groups were observed; this may have influenced the outcomes.

No blinding in assessing SSI within groups was adopted. The Centers for Disease Control (CDC) criteria [66] were used to assess presence of SSI however a part of subjectivity remains; therefore, blinding may have reduced the risk of bias.

NPWT unlikely causes harm, but its application lengthens intervention and costs are elevated. Cost evaluation must be attentively evaluated [47]. Each patient presenting SSI costs approximately $17,000 (in term of total amount) [67], while an iNPWT costs almost $250–500/day. However, it should be pointed out that increasing the SSI care bundle would be the first plan to be posed in action and it may result as effective in reducing dramatically SSI, without significant device and material costs. All additional techniques, material and devices may be evaluated as supplementary to the optimization of SSI care bundles. The same group who published one of the included RCT [53] performed even a retrospective trial [54]. Interestingly, the SSI rate in RCT and retrospective trial are similar in iNPWT group but is significantly different in the standard dressing groups. This data may confirm the necessity to improve SD before implementing a NPWT systematic use.

A similar phenomenon has been observed in meta-analysis. Strugala et al. [59] reported an SSI rate with SD that differs between retrospective and randomized trials (22.5% vs. 9.7%, respectively). In fact, whenever standardized dressing techniques are implemented, it usually results in a reduction in SSI rate. Adherence to SSI bundles has been shown to be one of the first and best system to reduce SSI.

A meta-analysis of cohort studies (patients before and after the implementation of SSI-management-bundles) analysed more than 8500 patients [68]. None of them implemented identical bundles but shared appropriate prophylaxis, hair removal, glycaemic control, and normothermia. Although the range of intervention was different, applying a bundle to wound care would reduce SSI rate from 15.1 to 7% (P = 000.5). Unfortunately, the overall compliance with guidelines for SSI attests around 40 to 60% [2, 69, 70]. Implementing adherence to bundles and guidelines will itself reduce SSI rate and then advanced dressing systems may be studied to further increase the achievement.

Two large trials on iNPWT are actually ongoing [71, 72]; however, none of them have IP as target population. RCT focusing on SSI reduction in immunocompromised patients are needed.

The use of surgical incision partial closure by distanced interrupted stitches in transplanted patients demonstrated very good results [36] and it may be considered to be applied in all emergency surgeries in IP or high-risk patients in order to reduce the SSI rate. This procedure represents a cost-effective and low-risk strategy in preventing SSI in patients operated for intrabdominal infections that as a matter of facts infect the surgical incision. The initial partial closure of the surgical incision may allow to proceed to complete closure in the early postoperative course once the risk of SSI has been reasonably cleared out.

Drain placement is debated and probably needs more investigation. In KT, the drain placement has always been done on patient-by-patient evaluation or by protocol of the different centres. Retrospective trials showed no advantage for drain placement [37] in reducing SSI. The retrospective nature of the studies may have led to underestimating the possibility that surgeons may have been more inclined to order imaging tests to detect potential fluid collection in the case that they have not placed a drain. Moreover, drains may have been probably placed more frequently in the high-risk population underestimating their benefit.

Patients on steroids experience impaired wound healing [73] and develop SSI more frequently in the postoperative period. However, high-dose corticosteroid administered for any reason, in acute setting, for less than 10 days preoperatively seems to not affect the incidence of SSI as shown in an RCT evaluating colonic resection [74]. Conversely, if chronic steroids assumption is examined, increased rates of SSI from 2- to 5-fold are reported [75].

Ismael et al. [76] conducted a large retrospective trial with 20,000 patients who underwent surgical procedure while on steroid therapy for more than 30 days. There was an increase of SSI (from 2.9 to 5% for superficial SSI) and a 4-fold increase in overall mortality; thus, this is probably reflecting a sicker population generally.

WHO guidelines for SSI suggest against the suspension of those drugs which may impair wound healing in a statement graded as conditional and with low quality of evidence [2]. The recommendation is based on a non-blinded RCTs from 1993 analysing 64 patients with rheumatoid arthritis on chronic methotrexate (MTX) randomized to 7 days MTX suspension before surgery (intervention) or normal assumption (control), undergoing orthopaedic procedures [77].

It has been demonstrated as Rapamycin inhibits early- and mid-stages angiogenesis, but not late-stage angiogenesis or lymph-angiogenesis [78]. This data may explain the effect on wound healing and subsequent SSI in patients undergoing emergency and general surgery while in immunomodulatory or immunosuppressive therapy.

No suggestion can be given regarding suspension of the immunomodulatory drug since evidence are lacking in favour or against it. However, surgeons must be aware of the possible complications and the various surgical strategies to reduce the risk.

This work presents as main strength the fact that it for the first time systematically analysed the existing literature about the topic and resumed all the data investigating SSI prevention and management in immunocompromised and high-risk patients. Moreover, it attempts to give precise indications to those who are in charge to manage such a frail patient.

As a counterpart, the present paper has several limitations: it lies in the proposal of finding an answer to a topic that carries multiple questions. There is no standardized definition of IP and it is difficult to find a single intervention that will lower the SSI rate in this population; therefore, the range of different topics included in our systematic review is wide. In many studies, IP are mixed to other cohorts and high-risk patients. In the existing literature, there are several RCTs on iNPWT, while other interventions are less investigated: there is no cunning in observing that economic and resource setting may influence the production of RCTs on iNPWT.

Applicability of the results is often sub-optimal given that few studies exist on IP-specific population and somehow is necessary to consider evidence derived from studies analysing similar cohorts of patients.

## Conclusion

Strict adherence to SSI infection preventing bundles must be implemented worldwide especially in immunocompromised patients. Partial skin closure can be used to reduce SSI in this population. A clear role of antibacterial prophylaxis in IPs should be ruled out. There is not sufficient evidence to definitively suggest in favour of prophylactic negative pressure wound therapy. The use of mTOR and CNI in transplanted patient needing ad emergent or undeferrable abdominal surgical procedure must be carefully and multidisciplinary evaluated. Lastly, it is necessary to elaborate a more widely approved definition of immunocompromised state. Without such shared definition, it will be hard to elaborate the needed methodologically correct studies for this fragile population.

## Data Availability

Not applicable

## Abbreviations

SSI: Surgical site infection
iNPWT: NPWT is applied to the surgical incisional wound with closed suture
HIV: Human immunodeficiency virus
AIDS: Acquired immunodeficiency syndrome
VEGF: Vascular endothelial growth factor
RCTs: Randomized controlled trials
LOS: Length of hospital stay
G-CSF: Granulocyte-colony stimulating factor
KT: Kidney transplanted
CD: Crohn’s disease
SSIRS: Surgical site infection risk score
MTX: Methotrexate
